# The Relationship Between Dietary Diversity and Mental Health Among Chinese Older Adults: Evidence from the Chinese Longitudinal Healthy Longevity Survey

**DOI:** 10.3390/nu18121936

**Published:** 2026-06-15

**Authors:** Shujuan Xiao, Xinru Li, Jiachi Zhang, Sihan Xu, Lei Shi, Xingcun Zhao

**Affiliations:** 1 School of Health Management, Guangzhou Medical University, Guangzhou 511495, China; 17853589587@163.com (S.X.); 18919032778@163.com (S.X.); 2 Key Research Base for Humanities and Social Sciences of Guangdong Higher Education Institutes, Guangzhou Medical University, Guangzhou 511495, China; 3 School of Public Health, Southern Medical University, Guangzhou 511495, China; 17861521071@163.com; 4 Department of Gerontology, Simon Fraser University, Vancouver, BC V6B 5K3, Canada; jiachi_zhang@sfu.ca; 5 Gloria Gutman Gerontology Research Centre, Simon Fraser University, Vancouver, BC V6B 5K3, Canada

**Keywords:** dietary diversity, mental health, sleep quality, self-rated quality of life, serial mediation, gender moderation

## Abstract

**Background:** Previous research has confirmed that dietary diversity is positively linked to mental health outcomes in older populations. Nevertheless, relevant evidence focusing specifically on Chinese older adults remains limited, and the internal mechanisms underlying this association I confirm. are not fully understood. Against this background, this study intended to investigate the association between dietary diversity and mental health among Chinese older individuals, explore the chain mediating roles of sleep quality and self-perceived quality of life, and further test whether gender moderates the above direct and mediating pathways. **Methods:** Using 2018 CLHLS data, 10,089 older adults aged 60 and above were selected as valid samples. Pearson correlation analysis was employed to determine the relationships between key variables. Hayes’ PROCESS macro Model 6 was used for baseline serial mediation analysis, and Model 85 was used for moderated serial mediation with gender as the moderator, adopting 5000 bootstrap samples. **Results:** The results revealed significant positive correlations (*p* < 0.01) between key variables, including dietary diversity, sleep quality, self-rated quality of life, and mental health. Model 6 showed that dietary diversity serves as a positive and significant predictor of mental health (B = 0.130, *p* < 0.001). Three significant mediating pathways were identified through which dietary diversity affects mental health: (1) sleep quality (B = 0.076, 95% CI: 0.062, 0.092), (2) self-rated quality of life (B = 0.100, 95% CI: 0.083, 0.118), and (3) sleep quality and self-rated quality of life (B = 0.020, 95% CI: 0.016, 0.025). The total mediating effect of the three pathways reached 59.94%. Model 85 found that the interaction term of dietary diversity x gender was non-significant (*p* > 0.05), demonstrating no statistically significant gender moderation of any pathway. Gender-stratified conditional effects revealed numerical differences across subgroups. **Conclusions:** Higher dietary diversity is significantly correlated with better mental health among Chinese older adults. Sleep quality and self-rated quality of life play significant roles as serial mediators in this association. Although gender does not statistically moderate the whole association mechanism, subtle gender heterogeneity exists in the pathway effect magnitude. The above findings offer novel insights into the underlying mechanisms. Strategies aimed at improving dietary diversity, combined with targeted interventions to enhance sleep quality and self-rated quality of life, with slight gender-differentiated auxiliary suggestions, may effectively promote mental health and contribute to active aging in later life.

## 1. Introduction

Global demographic aging is reshaping population structures worldwide and posing considerable challenges to public health systems [1]. Official data released by the National Bureau of Statistics show that the number of people aged 60 and above in China is expected to reach 323.38 million by the end of 2025, accounting for 23.0% of the total population. The aging population has entered a rapid growth phase [2]. As global population aging accelerates, mental health issues are emerging as a critical public health concern among older adults. Approximately 20% of people aged 60 and above have mental or neurological disorders [3]. Under the dual backdrop of increased population aging and rising chronic health risks, the mental well-being of older individuals has become a core issue in public health and healthy aging. Depression and other psychological symptoms have a high incidence and are strongly concealed among older adults, which not only significantly reduces their ability to take care of themselves and their quality of life but also increases the risk of chronic disease deterioration, disability [4], and even death [5]. The Healthy China 2030 initiative emphasizes strengthening mental health services for older adults and improving the intervention system for modifiable health determinants. In this context, identifying modifiable, low-cost, and protective factors for mental health has important theoretical value and practical significance.

Dietary nutrition is a fundamental determinant that maintains the body’s homeostasis, affects brain function, and regulates emotions. In recent years, Nutritional Psychiatry has rapidly developed as an interdisciplinary field [6]. A growing body of evidence indicates that dietary structure, rather than a single nutrient, is the key determinant of psychological adaptation and emotional health [7,8,9]. Dietary diversity, as a core indicator for measuring dietary quality, reflects the richness of food intake and directly relates to whether the intake of macronutrients and micronutrients is balanced [10]. As a modifiable determinant of health, the relationship between dietary nutrition and mental health is receiving increasing attention. A prospective study shows a robust positive correlation between dietary diversity and the mental health of older adults [11]. Further longitudinal studies have revealed a positive relationship between dietary diversity and subjective well-being, providing evidence for nutritional interventions to improve mental health in later life [12]. The potential mechanism may involve a diverse diet that guarantees the supply of B vitamins, omega-3 fatty acids, tryptophan, and antioxidants. By regulating neurotransmitter synthesis, reducing neuroinflammation, and improving hypothalamic–pituitary–adrenal (HPA) axis function, such a diet directly maintains brain homeostasis and emotional regulation ability [13]. Multinational studies have shown that high dietary diversity is significantly associated with lower rates of depression and anxiety, improved emotional regulation, enhanced subjective well-being, and improved cognitive function [14].

Despite consistent evidence linking dietary diversity to mental health, the underlying mechanisms have not been sufficiently clarified in Chinese older adults. Exploring these mechanisms is crucial for developing targeted preventive strategies. In this mechanism, two possible mediators are sleep quality and self-rated quality of life. Sleep quality is considered a key physiological mediator variable in the potential pathway between diet and mental health. Dietary diversity is positively associated with sleep quality, and a lack of dietary diversity may lead to poor sleep quality [15]. A study on the older population in Japan confirmed that a diet consisting of multiple food groups was more beneficial to sleep quality than a diet limited to a single type of food among older adults [16]. A balanced and diverse diet can provide tryptophan, melatonin precursors, B vitamins, and minerals, improving difficulty falling asleep, sleep maintenance disorders, and sleep structure disorders [17,18]. There is robust evidence indicating that sleep quality is a critical factor affecting the mental health of older adults [19]. Sleep deprivation or poor sleep quality can trigger excessive activation of the HPA axis, increase inflammation levels, and decrease emotional regulation ability, thereby inducing or exacerbating psychological problems [20]. Dietary interventions have been proven effective in auxiliary treatment and self-management of sleep disorders and mental health problems [21]. Meanwhile, self-rated quality of life reflects a subjective evaluation of living status [22]. More importantly, it is an intermediary bridge connecting sleep and psychological state. Specifically, prior research found that sleep quality was significantly linked to higher quality of life [23,24], and higher quality of life can strengthen mental health [25,26]. A higher quality of life means stronger psychological adaptability, more positive coping strategies [27], and lower levels of negative emotions, which can significantly protect mental health. This suggests that sleep quality and self-rated quality of life may function as serial mediators in the relationship between dietary diversity and mental health. However, this integrated mechanism has not been empirically tested in older adults, and most studies focus on single mediators rather than serial pathways. In addition, there are gender differences in physiological endocrinology, dietary habits, sleep sensitivity, and psychological coping mechanisms [28,29,30]. Numerous epidemiological studies, both domestic and international, have documented gender disparities in the prevalence of depression and nutritional absorption efficiency among older adults. Prior research identified a distinct depression prevalence across genders [31]. The existing literature consistently identifies statistically significant gender disparities in dietary diversity levels among older adults in Bangladesh [32]. Distinguishing gender differences can provide empirical evidence for targeted nutritional interventions in the community; therefore, this study incorporates gender as a moderator variable and conducts a moderated chain mediation analysis.

**Figure 1:**
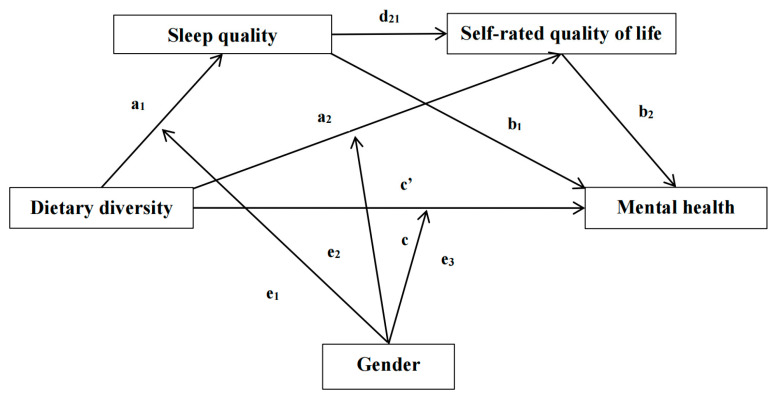
The hypothesized moderated serial mediation model illustrating the relationship between dietary diversity and mental health, in which sleep quality and self-rated quality of life function as serial mediators, and gender acts as a moderator. Note: a, b, c, c’, d, and e represent path coefficients.

Accordingly, the present study aimed to examine the serial mediating effects of sleep quality and self-rated quality of life, as well as the moderating effect of gender linking dietary diversity to mental health, among Chinese older adults. In summary, five research hypotheses are proposed in the present study (Figure 1).

**H1.**  *Dietary diversity can significantly positively predict mental health in older adults.*

**H2.**  *Sleep quality exerts an independent mediating effect on the association between dietary diversity and mental health.*

**H3.**  *Self-rated quality of life plays an independent mediating role in the association between dietary diversity and mental health.*

**H4.**  *Sleep quality and self-rated quality of life play a chain mediating role in the association between dietary diversity and mental health.*

**H5.**  *Gender serves as a moderator in both the direct and indirect mechanisms by which dietary diversity influences older adults’ mental health. The magnitudes of direct and mediated effects vary dramatically between male and female older adults.*

Using data from a large sample of 10,089 older people in Chinese communities, the current study aimed to clarify direct and indirect effects and further verify the moderating difference across gender groups, providing evidence for nutritional, sleep, and quality-of-life interventions to promote mental health in older adults.

## 2. Materials and Methods

### 2.1. Study Design and Participants

This study used publicly accessible data from the 2018 wave of the Chinese Longitudinal Healthy Longevity Survey (CLHLS). To ensure scientific rigor and national representativeness, the CLHLS used a multistage disproportional targeted random sample technique that included 23 provinces, municipalities directly under the Central Government, and autonomous regions throughout China. CLHLS was approved by the Research Ethics Boards of Peking University and Duke University (IRB00001052-13074). All enrolled participants provided written informed consent.

**Figure 2:**
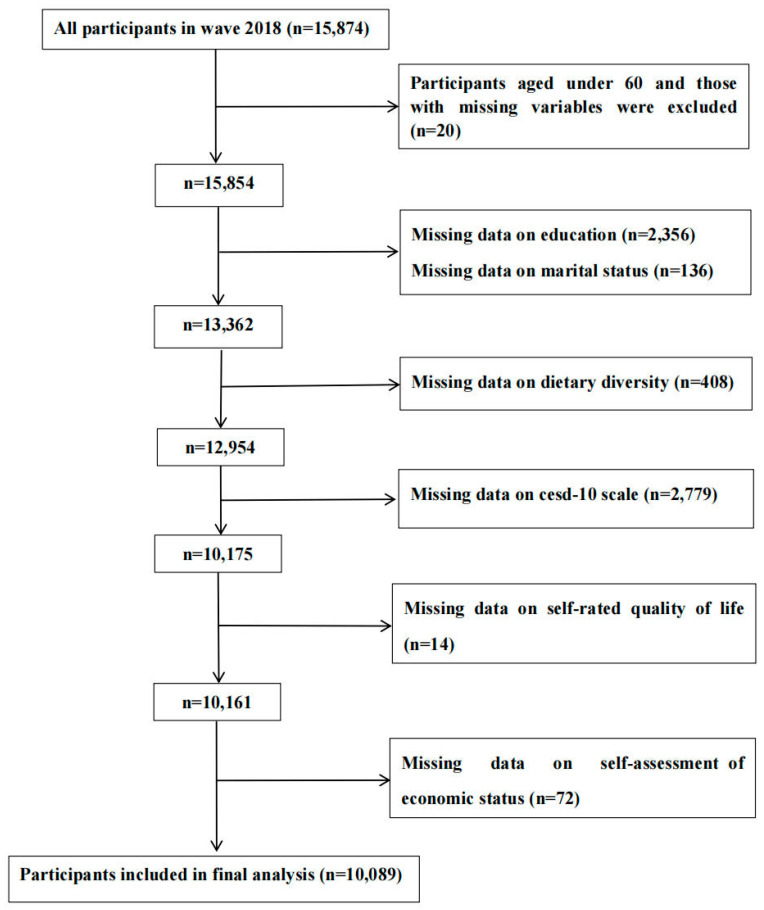
Flowchart of the study sample.

The research subjects were restricted to older individuals aged 60 years and above with complete information on key variables. After excluding individuals with missing key data, 10,089 participants were included. The sample flowchart is shown in Figure 2.

### 2.2. Materials

#### 2.2.1. Dietary Diversity

Referring to previous studies [33], the food consumption frequency questionnaire in CLHLS was used to calculate the Dietary Diversity Score (DDS), which includes the intake of 9 foods, including fruits, vegetables, meat, eggs, fish, beans, nuts, milk, and tea. Fruits and vegetables have four options: “every day/almost every day”, “often”, “occasionally”, and “rarely or never”. An answer of “every day/almost every day” or “often” is rated 1 point, while an answer of “occasionally” or “rarely or never” is rated 0 points. Meat, eggs, fish, beans, nuts, milk, and tea have five options: “almost every day”, “not every day, but at least once a week”, “not every week, but at least once a month”, “not every month, but occasionally”, and “rarely or never”. An answer of “almost every day” or “not every day, but at least once a week” receives 1 point; otherwise, the value is 0. The overall DDS is the sum of scores for all nine food items, with a total scoring range of 0 to 9. Higher total scores represented more diversified daily dietary structures. The DDS scale has acceptable reliability (Cronbach’s alpha = 0.666) and validity (KMO = 0.768, *p* < 0.001).

#### 2.2.2. Mental Health

Referring to the previous literature [34], the 9-item abbreviated version of the Center for Epidemiologic Studies Depression Scale (CESD-9) was employed to assess mental health status. Using a 5-point Likert scale for responses, the answer range is 1–5: 1 = “always”, 2 = “often”, 3 = “sometimes”, 4 = “seldom”, 5 = “rarely or never”. At the same time, two positive items are assigned scores in reverse. The result range is 9–45, with higher scores indicating lower levels of depression and better mental health. In this study, the CESD-9 scale had good reliability (Cronbach’s alpha = 0.806).

#### 2.2.3. Sleep Quality

Sleep quality was assessed with a single item: “How is your sleep quality now?” Responses were scored reversely: 1 = “very bad”, 2 = “bad”, 3 = “so so”, 4 = “good”, 5 = “very good”. This questionnaire item exhibited good reliability and has been widely validated and applied to older adults [35,36].

#### 2.2.4. Self-Rated Quality of Life

The self-rated quality of life of older adults was evaluated by assessing their response to the question “How do you feel about your current life?” Using a 5-point Likert scale for responses, the answer range is 1–5: 1 = “very bad”, 2 = “bad”, 3 = “so so”, 4 = “good”, 5 = “very good”. The higher the score, the higher the self-rated quality of life of the older adult. This single item exhibited good reliability and has been widely validated and applied to older adults [37,38].

#### 2.2.5. Measurements of Other Variables

Covariates were selected based on previous studies [39,40]. The control variables included age, education level, self-assessment of economic status, residence area, and marital status. In addition, gender was set as a moderating variable in our subsequent statistical analyses to examine potential heterogeneous associations across male and female groups.

### 2.3. Statistical Analysis

All statistical analyses in this study were performed using IBM SPSS 26.0 software. Pearson correlation analysis was conducted to examine the correlations between all core research variables. To clarify the interrelationships and underlying mechanisms among dietary diversity, mental health, sleep quality, and self-rated quality of life, mediation analysis was carried out using the PROCESS macro 4.2 for SPSS, which was developed by Hayes. Specifically, Model 6 of the multiple mediation analysis was adopted. A total of 5000 bootstrap resamples were generated to calculate 95% bias-corrected confidence intervals (CIs). An indirect mediating effect was statistically significant when the 95% CI did not contain zero. This macro enabled the calculation and testing of direct effects, total effects, and indirect effects of dietary diversity on mental health. In order to determine whether gender significantly moderates the direct path and mediating paths in the entire model, Model 85 of the moderated serial mediation analysis was further constructed, adding the interaction item of dietary diversity x gender to each regression equation.

## 3. Results

### 3.1. Basic Characteristics of the Sample

The valid sample of this study comprised a total of 10,089 Chinese older adults, including 4598 males (45.57%) and 5491 females (54.43%). In terms of age distribution, 4161 participants were aged 60–79 years, 4668 were aged 80–99 years, and 1260 were over the age of 100. Regarding educational attainment, 2181 people had completed junior high school or higher education, 3470 had completed primary school, and 4438 were illiterate. In terms of self-reported economic status, the majority of older persons (7058) reported a medium economic level, while 2062 participants regarded themselves as rich, and 969 participants rated their economic status as poor. There were 4187 participants who lived in rural regions, 3322 who lived in towns, and 2580 who lived in urban areas. A total of 4561 participants were currently married and living with spouses, whereas 5224 were widowed, with a small number of separated, divorced, and never-married older adults. As shown in Table 1, older adults’ mental health levels were significantly different across gender, age group, educational level, self-assessed economic status, residential area, and marital status (*p* < 0.001).

### 3.2. Common Method Bias

To preclude common method bias from undermining the reliability of the study results, Harman’s single-factor test was adopted for detection. This method is widely applied in common method bias assessment [41,42]. The exploratory factor analysis results indicated that the maximum variance explained by the first extracted component was 22.088%, which was far lower than the conventional critical cutoff value of 40% [41]. Thus, it was confirmed that no serious common method bias existed in the current study dataset.

### 3.3. Descriptive Statistics and Correlation Analysis

The results of all core research variables based on the 10,089 valid samples are presented in Table 2, including mean values and standard deviations and Pearson correlation coefficients. The means and standard deviations (M ± SD) of dietary diversity, sleep quality, self-rated quality of life, and mental health were (4.793 ± 2.021), (3.518 ± 0.980), (3.922 ± 0.798), and (34.283 ± 5.715), respectively. Pearson correlation analyses of the key variables are also detailed in Table 2. Dietary diversity was positively correlated with sleep quality (*r* = 0.149, *p* < 0.01), self-rated quality of life (*r* = 0.208, *p* < 0.01), and mental health (*r* = 0.185, *p* < 0.01). Sleep quality was positively correlated with self-rated quality of life (*r* = 0.259, *p* < 0.01) and mental health (*r* = 0.333, *p* < 0.01). Self-rated quality of life was positively correlated with mental health (*r* = 0.398, *p* < 0.01). The preliminary results support the existence of an intrinsic correlation between variables, which can be further tested for mediating effects.

### 3.4. Moderated Serial Mediation Model

This study utilized the PROCESS Model 85 to construct a moderated chain mediation model with gender as the moderator and sleep quality and self-rated quality of life as chain mediators. The analysis included age, marital status, place of residence, educational attainment, and self-rated economic status as control variables.

As shown in Table 3, the results indicated that dietary diversity positively predicted sleep quality in older adults (B = 0.043, *p* < 0.01); the regression coefficient for gender was significantly negative (B = −0.255, *p* < 0.001), but the interaction coefficient between dietary diversity and gender did not reach statistical significance (B = 0.010, *p* > 0.05), indicating that gender does not act as a moderator in the pathway through which dietary diversity influences sleep quality.

Dietary diversity was found to positively enhance self-rated quality of life (B = 0.045, *p* < 0.001), and sleep quality also had a significant positive effect on self-rated quality of life (B = 0.166, *p* < 0.001); neither the main effect of gender nor the interaction term between dietary diversity and gender was statistically significant (*p* > 0.05).

After controlling for confounding variables, dietary diversity continued to significantly and positively predict mental health levels (B = 0.212, *p* < 0.001); the two mediating variables, sleep quality (B = 1.313, *p* < 0.001) and self-rated quality of life (B = 2.129, *p* < 0.001), also exerted positive effects on mental health. At the same time, neither the main effect of gender nor the dietary diversity × gender interaction term was significant (*p* > 0.05). Overall, gender does not exert a moderating effect on the direct path from dietary diversity to mental health or on any of the mediating paths.

**Figure 3:**
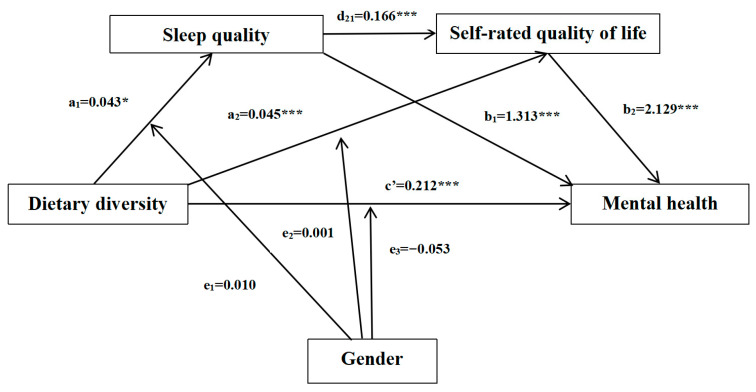
The hypothesized moderated serial mediation model illustrating the relationship between dietary diversity and mental health, in which sleep quality and self-rated quality of life function as serial mediators, and gender acts as a moderator. Path coefficients are shown. Note: * *p* < 0.05, *** *p* < 0.001.

The pathway of the model is depicted in Figure 3. Path coefficients demonstrated that all relationships within the model were significantly positive. Even after incorporating sleep quality and self-rated quality of life as mediators, the direct effect of dietary diversity on mental health remained significant, suggesting that the association between dietary diversity and mental health is partially mediated by these two variables. The interaction term between dietary diversity and gender did not reach statistical significance for any path (all *p* > 0.05), indicating that gender did not significantly moderate the direct association between dietary diversity and mental health or the mediating pathways.

### 3.5. Bootstrap Test of Mediators

In the bootstrap test, a mediating effect was deemed significant if the 95% confidence interval (CI) of the path coefficient did not contain zero [43]. Table 4 presents the total, direct, and indirect effects of dietary diversity on mental health. All paths yielded 95% CIs excluding zero, confirming their significance. According to Model 6’s analytical outcomes, the total effect of dietary diversity on mental health was 0.327, with a direct predictive effect of 0.130, accounting for 39.76% of the total effect. The overall indirect mediating effect was 0.196, which explained 59.94% of the total association between dietary diversity and mental health. After incorporating sleep quality and self-rated quality of life as mediators, the direct effect of dietary diversity on mental health remained significant (c’ = 0.130, *p* < 0.001). Three significant indirect pathways were identified: (1) sleep quality (B = 0.076, 95% CI: 0.062, 0.092), representing 23.24% of the total effect, (2) self-rated quality of life (B = 0.100, 95% CI: 0.083, 0.118), accounting for 30.58% of the total effect, and (3) sleep quality and self-rated quality of life (B = 0.020, 95% CI: 0.016, 0.025), contributing 6.12% to the total effect. The cumulative proportion of the three mediating pathways reached 59.94%.

Model 85 was used to examine differences in path effects across gender groups. Stratified analysis by gender (Table 5) showed that the direct and all indirect effects remained statistically significant for both males and females, with slight disparities in effect sizes between the two gender subgroups. Dietary diversity had a significant direct predictive effect on the mental health of both male and female older adults, with effect sizes of 0.159 and 0.106, respectively, and confidence intervals for both that did not cross zero. In the single sleep mediation path, the indirect effect size for female older adults (B = 0.081) was slightly higher than that for male older adults (B = 0.068). In the independent mediation path of self-rated quality of life, the effect levels for men and women were nearly identical, at 0.099 for men and 0.101 for women; in the chained mediation path, the effect size for women (B = 0.022) was also slightly greater than that for men (B = 0.018).

All pathways remained statistically significant for both genders. Although the overall moderating effect was not significant, there were slight differences in effect sizes across the various mediation pathways between the genders, suggesting that the mental health benefits of dietary diversity exhibit some gender heterogeneity in terms of effect strength.

## 4. Discussion

This study is based on a large sample of 10,089 older adults in communities across the country and systematically explores the impact of dietary diversity on mental health and its underlying mechanisms. The results revealed that dietary diversity is positively associated with mental health in Chinese older adults, both directly and through the serial mediating pathways of sleep quality and self-rated quality of life. At the overall level, gender did not exert a significant moderating effect, but there were slight differences between men and women in the values of the effects along each path. These findings provide novel insights into the nutritional and psychological mechanisms involved in late-life mental health and offer high-quality epidemiological evidence for the promotion of healthy aging.

### 4.1. The Direct Protective Effect of Dietary Diversity on Mental Health

This study found that, after controlling for demographic confounding factors, dietary diversity can significantly and positively predict the mental health of older people (B = 0.130, *p* < 0.001), with a total effect value of 0.327. Therefore, hypothesis H1 was supported: higher dietary diversity was associated with better mental health. This result aligns with the mainstream conclusions in the field of international nutritional psychiatry. A meta-analysis by Nazarian et al. [14] demonstrated that the higher the dietary diversity, the lower the risk of depression and depressive symptoms, supporting the robustness of this relationship. A varied diet provides a more comprehensive range of nutrients, which helps to maintain normal brain function and regulate mood, thereby improving mental health [44]. Therefore, improving dietary diversity has more prominent public health value in preventing psychological problems. Dietary diversity is a modifiable, low-cost protective factor suitable for community-based health management.

### 4.2. Independent Mediating Effect of Sleep Quality on Dietary Diversity and Mental Health

This study confirms that sleep quality has a strong independent mediating effect on the association between dietary diversity and mental health, with an effect rate of 23.24%, supporting H2. The mediation may be related to the influence of diet on blood glucose levels, tryptophan, and melatonin precursor supply. Dietary diversity improves sleep quality overall by providing nutrients such as tryptophan, melatonin precursors, calcium, magnesium, and B vitamins, which improve sleep latency, reduce nighttime awakenings, and increase the proportion of deep sleep [45,46,47]. Stable blood sugar levels help reduce nighttime awakenings and improve sleep quality [48]. Specifically, tryptophan acts as the core precursor for serotonin and melatonin synthesis, which helps regulate circadian rhythms and improve sleep [49]. Melatonin is directly involved in the process of sleep regulation [50]. Active attention should be paid to improving older persons’ sleep quality, strengthening nutritional guidance to improve dietary diversity, and reducing sleep disturbances, thereby promoting their mental health.

### 4.3. Independent Mediating Role of Self-Rated Quality of Life

This study found that the mediating effect of self-rated quality of life is the strongest, accounting for 30.58% of the total effect, higher than that explained by sleep quality. Thus, hypothesis H3 is supported: dietary diversity mainly protects the mental health of older adults by improving their subjective life evaluation. This discovery is highly consistent with the subjective well-being theory of health psychology [51], which posits that higher life satisfaction buffers stress and reduces vulnerability to psychological disorders. Diversified diets can provide a richer dining experience and increase the joy of and satisfaction with life. Good nutritional status also helps to improve an individual’s physical and social functioning [52,53], thereby enhancing their self-rated quality of life. The improvement of self-rated quality of life helps to enhance individuals’ sense of happiness and belonging, thereby improving their mental health level [51]. Subjective quality of life, as a cognitive–emotional integration indicator, can amplify the psychological benefits of healthy behaviors. This study further confirms that quality of life is the core subjective pathway connecting diet and mental health in the context of Chinese culture, suggesting that interventions should not only focus on objective nutritional improvement but also pay attention to the improvement of quality of life among older adults.

### 4.4. Chain Mediation Mechanism of Sleep Quality and Self-Rated Quality of Life

The association between dietary diversity and mental health is jointly regulated by the chain mediation effect of sleep quality and self-rated quality of life, with the mediating effect accounting for 6.12% of the total effect. This result supports Hypothesis H4: older adults with higher dietary diversity are more likely to have better mental health through improved sleep quality and self-rated quality of life. Our findings enrich the relevant literature by confirming the serial mediating role of sleep quality and self-rated quality of life in the relationship between dietary diversity and mental health. Special attention should be paid to older individuals with low dietary diversity who simultaneously suffer from poor sleep quality and low self-rated quality of life. Older adults with higher dietary diversity often have better sleep quality [15], which helps improve daytime functioning and emotional stability [54]. Better daytime functioning and emotional stability can enhance social participation and role adaptation [55], thereby improving self-rated quality of life. Older adults with higher self-rated quality of life may still be at increased risk of poor mental health. This chain mediation pathway fully reflects the Biopsychosocial Model [56], wherein objective health behaviors (diet) influence physiological states (sleep), which in turn shape subjective evaluations (quality of life) and psychological outcomes.

### 4.5. Gender Moderation Test and Subgroup Heterogeneity Analysis

Model 85 results show that the dietary diversity × gender interaction term was not statistically significant in any of the equations (*p* > 0.05) and overall does not support Hypothesis H5. However, numerical heterogeneity was observed in the effects of subgroup conditions: women derived greater benefits from single-mediation and chain mediation models related to sleep, whereas the mediating effects on quality of life were comparable between men and women. Older women’s sleep regulatory system is more vulnerable to insufficient nutrient intake. Hence, dietary diversification leads to a more prominent sleep improvement. Meanwhile, females are more sensitive to daily dining experiences [28], which accelerates the cascade improvement of sleep–life satisfaction and mental health. Physiologically, hormonal fluctuations in women make sleep more susceptible to disruption caused by nutritional deficiencies [29,57], resulting in more pronounced sleep benefits from dietary improvements. The results can serve as a reference for targeted interventions.

### 4.6. Theoretical and Practical Significance

On a theoretical level, this study clarifies the contribution ratios of direct effects and three indirect effects and expands the mechanistic framework of the relationship between nutrition and psychological health. Firstly, dietary guidelines are an important aspect of health intervention programs among older adults. In particular, the government should focus on incorporating the improvement of dietary diversity into policies to promote mental health and strengthen awareness of sustainable diets. Secondly, family members, social networks, and resources should be mobilized to assist in promoting nutritional orientation, encouraging sleep regulation, and improving quality of life as part of public health measures to fully support older adults. Thirdly, targeted intervention strategies focusing on sleep quality and self-rated quality of life are essential for improving the mental well-being among older adults. For older adults with sleep problems or low quality of life, targeted support should be provided to help them handle health challenges correctly and seek professional assistance. Fourthly, and most importantly, greater public health attention should be paid to older adults with low dietary diversity. In clinical and community health practice, proactive intervention mechanisms should be established to realize the early screening and detection of potential health risks among older adults. Government departments and community organizations need to strengthen health management services, regularly conduct nutritional examinations and psychological health assessments for older adults, and provide targeted intervention measures.

## 5. Research Limitations and Prospects

Several limitations of this study need to be acknowledged. Firstly, due to the cross-sectional design of this study, causal relationships between dietary diversity, sleep quality, self-rated quality of life, and mental health cannot be established. Future longitudinal studies and randomized controlled trials are needed to verify causal effects. Secondly, the use of self-reported data may introduce measurement bias. Future research should consider combining objective nutritional indicators and sleep data from wearable devices to improve validity. Thirdly, only gender was examined as a potential moderator in the present study. In the future, the moderating effects of factors such as age, income, and urban–rural status can be explored to develop precisely classified dietary intervention schemes for heterogeneous subgroups.

## 6. Conclusions

This study systematically analyzed the interrelationships and internal mechanisms between dietary diversity, sleep quality, self-rated quality of life, and mental health among Chinese older adults. Dietary diversity was found to significantly and positively predict mental health in older adults, with sleep quality and self-rated quality of life acting as chained multiple mediators, with the total indirect effect accounting for 59.94%. No significant overall moderating effect of gender was observed for any pathway, but modest numerical heterogeneity existed in mediating effect sizes between male and female seniors, with females benefiting more from a diversified diet through indirect sleep-related paths. Dietary diversity should be prioritized in national health interventions. Through nutrition education, the public should be guided to diversify their food choices and optimize their dietary patterns, thereby laying a solid physiological foundation for mental health. At the same time, dietary guidance should be integrated with sleep health management, using dietary improvements to enhance sleep quality and break the chain of damage to mental health caused by dietary imbalances and poor sleep. Furthermore, efforts to improve nutrition, optimize sleep, and enhance self-reported quality of life should be advanced in a coordinated manner to establish a positive feedback loop. Targeted health management interventions specifically targeting older adults can effectively improve their mental health, thus contributing to the achievement of healthy aging.

## Figures and Tables

**Table 1:** Basic characteristics of the sample associated with mental health (n = 10,089).

Variables	n (%)	CESD-9 (M ± SD)	*t*/*F*	*p*
Gender			9.488	<0.001 ^a^
Male	4598 (45.57)	34.868 ± 5.575		
Female	5491 (54.43)	33.793 ± 5.785		
Age			51.765	<0.001 ^b^
60–79	4161 (41.24)	34.961 ± 5.624		
80–99	4668 (46.27)	33.874 ± 5.741		
≥100	1260 (12.49)	33.562 ± 5.692		
Education level			129.533	<0.001 ^b^
Illiteracy	4438 (43.99)	33.309 ± 5.700		
Primary school	3470 (34.39)	34.761 ± 5.546		
Junior high school and above	2181 (21.62)	35.504 ± 5.683		
Self-assessment of economic status			344.929	<0.001 ^b^
Rich	2062 (20.44)	36.281 ± 5.121		
Medium	7058 (69.96)	34.198 ± 5.520		
Poor	969 (9.60)	30.647 ± 6.374		
Residence area			23.510	<0.001 ^b^
City	2580 (25.57)	34.923 ± 5.827		
Town	3322 (32.93)	33.928 ± 5.680		
Rural	4187 (41.50)	34.171 ± 5.642		
Marital status			43.118	<0.001 ^b^
Currently married and living with spouse	4561 (45.21)	35.064 ± 5.531		
Separated	190 (1.88)	34.095 ± 5.586		
Divorced	33 (0.33)	33.546 ± 5.316		
Widowed	5224 (51.78)	33.658 ± 5.769		
Never married	81 (0.80)	31.358 ± 6.940		

Note: ^a^ *t*, *t*-test; ^b^ *F*, one-way ANOVA.

**Table 2:** Descriptive statistics and correlation coefficients of key variables (n = 10,089).

Variables	M ± SD	1 Dietary Diversity	2 Sleep Quality	3 Self-Rated Quality of Life	4 Mental Health
1 Dietary diversity	4.793 ± 2.021	1			
2 Sleep quality	3.518 ± 0.980	0.149 **	1		
3 Self-rated quality of life	3.922 ± 0.798	0.208 **	0.259 **	1	
4 Mental health	34.283 ± 5.715	0.185 **	0.333 **	0.398 **	1

Note: M = mean, SD = standard deviation, ** *p* < 0.01.

**Table 3:** Regression coefficients for the moderated serial mediation model (n = 10,089).

Criterion	Predictors	R^2^	F	B	t	95% CI
Sleep quality	Dietary diversity	0.053	70.047	0.043 **	2.731	(0.012, 0.073)
	Gender	-	-	−0.255 ***	−5.038	(−0.354, −0.156)
	Dietary diversity × Gender	-	-	0.010	0.993	(−0.009, 0.028)
Self-rated quality of life	Dietary diversity	0.155	206.072	0.045 ***	3.775	(0.022, 0.069)
	Sleep quality	-	-	0.166 ***	21.721	(0.151, 0.181)
	Gender	-	-	0.071	1.821	(−0.005, 0.147)
	Dietary diversity × Gender	-	-	0.001	0.159	(−0.013, 0.016)
Mental health	Dietary diversity	0.246	329.114	0.212 ***	2.610	(0.053, 0.371)
	Sleep quality	-	-	1.313 ***	24.760	(1.209, 1.417)
	Self-rated quality of life	-	-	2.129 ***	31.595	(1.997, 2.261)
	Gender	-	-	0.100	0.381	(−0.416, 0.616)
	Dietary diversity × Gender	-	-	−0.053	−1.073	(−0.150, 0.044)

Note: ** *p* < 0.01, *** *p* < 0.001. Age, education level, self-assessment of economic status, residence area, and marital status were analyzed as control variables.

**Table 4:** Indirect effects of dietary diversity on mental health (Model 6).

Model Pathways	B	Boot SE	Effect Size	95% CI
Total effect	0.327 ^a^	0.030	100%	(0.268, 0.386)
Direct effect	0.130 ^a^	0.028	39.76%	(0.076, 0.185)
Total indirect effect	0.196 ^a^	0.013	59.94%	(0.171, 0.223)
Dietary diversity → sleep quality → mental health	0.076 ^a^	0.008	23.24%	(0.062, 0.092)
Dietary diversity → self-rated quality of life → mental health	0.100 ^a^	0.009	30.58%	(0.083, 0.118)
Dietary diversity → sleep quality → self-rated quality of life → mental health	0.020 ^a^	0.002	6.12%	(0.016, 0.025)

Note: ^a^ Empirical 95% confidence interval does not overlap with zero.

**Table 5:** Conditional direct and indirect effects of X on Y by gender (Model 85).

Model Pathways	Gender	B	Boot SE	95% CI
Direct effect				
	Male	0.159 ^a^	0.039	(0.083, 0.235)
	Female	0.106 ^a^	0.036	(0.036, 0.176)
Dietary diversity → sleep quality → mental health				
	Male	0.068 ^a^	0.010	(0.049, 0.089)
	Female	0.081 ^a^	0.010	(0.062, 0.101)
Dietary diversity → self-rated quality of life → mental health				
	Male	0.099 ^a^	0.013	(0.074, 0.124)
	Female	0.101 ^a^	0.012	(0.079, 0.124)
Dietary diversity → sleep quality → self-rated quality of life → mental health				
	Male	0.018 ^a^	0.003	(0.013, 0.024)
	Female	0.022 ^a^	0.003	(0.016, 0.028)

Note: ^a^ Empirical 95% confidence interval does not overlap with zero.

## Data Availability

The data for this study are available from the Chinese Longitudinal Healthy Longevity Survey (CLHLS), conducted by the Peking University Research Center for Healthy Aging and Development. We have also obtained authorization to use this data for our research.
